# The Outcome of the Use of Continuous Action Compression Device for the First Metatarsophalangeal Joint Fusion

**DOI:** 10.7759/cureus.74168

**Published:** 2024-11-21

**Authors:** Ahmed Ismail, Gary Hannant, Ahmed Ashour, Damian Broadhurst

**Affiliations:** 1 Orthopaedics, Bradford Royal Infirmary, Bradford, GBR; 2 Orthopaedics and Trauma, Bradford Royal Infirmity, Bradford, GBR; 3 Trauma and Orthopaedics, Queen Elizabeth Hospital Birmingham, Birmingham, GBR; 4 Surgery, Sheffield Children's NHS Foundation Trust, Sheffield, GBR

**Keywords:** ankle and foot, foo and ankle, great toe, mtp fusion, small joint arthritis

## Abstract

Introduction: First metatarsophalangeal (MTP) joint fusion is a widely accepted surgical intervention for treating severe arthritis, deformities, and instability of the first MTP joint. This paper provides a review of a single surgeon's experience with continuous compression implants (CCI), which offer a notable advantage by providing uniform compression across a larger surface area of the fusion site compared to plate and screw constructs. This design potentially reduces soft tissue irritation and, consequently, the need for subsequent implant removal. It also saves on cost and has the potential to reduce the length of surgery.

Methods: A retrospective review was conducted on 27 patients (n=36 feet) who underwent primary first metatarsophalangeal joint (MTPJ) fusion using continuous compression implants (CCI) between March 2020 and April 2024 at Bradford Royal Infirmary. Patient data were collected from the surgeon's logbook and medical records. The outcomes analyzed included the fusion rate and complications. Statistical analysis was performed using SPSS version 22.0, with p<0.05 considered significant.

Results: The mean age of the cohort was 60.24 years (range 41-90), with 88.88% female. The ratio of left to right was 70%. The mean follow-up duration was 27 months (range 6-48 months). Complete fusion of the first MTPJ was achieved in 34 out of 36 feet (94.4%). Nonunion occurred in one patient, while delayed union was observed in another. Clinically, 35 out of 36 patients (97.3%) reported satisfaction with the procedure, with one patient requiring metalwork removal and revision due to loosening.

Conclusion: Early results show that the rate of fusion achieved by using the CCI for the first MTPJ arthrodesis in our series was comparable to that of other devices quoted in the literature.

## Introduction

The first metatarsophalangeal (MTP) joint is essential for normal foot function, particularly in gait and weight-bearing activities. Pathologies such as hallux rigidus, severe hallux valgus, and rheumatoid arthritis can severely impair joint function, leading to pain and disability. First, MTP joint fusion, or arthrodesis, is a surgical procedure designed to permanently join the bones of the joint, thereby alleviating pain and restoring function. This paper reviews the indications, surgical techniques, outcomes, and complications associated with the first MTP joint fusion, providing a comprehensive understanding of this procedure [1].

Indications and patient selection

First, MTP joint fusion is indicated for patients with severe arthritis, deformity, or instability that has not responded to conservative treatments. Hallux rigidus, characterized by degenerative arthritis of the first MTP joint, is a common indication for this procedure [2,3]. Patients with rheumatoid arthritis or severe hallux valgus also benefit from fusion to relieve pain and improve function [4].

Surgical techniques

Various techniques for the first MTP joint fusion have been described, including dorsal plate fixation, crossed screw fixation, and cup-and-cone reaming. Dorsal plate fixation is preferred for its stability and ease of application. A study by Coughlin et al. [3] demonstrated that dorsal plate fixation with an interfragmentary screw provides strong fixation and high union rates. Another technique involves the use of crossed screws, which offers a biomechanically stable construct but can be technically demanding [4,5]. The choice of technique often depends on surgeon preference and specific patient factors.

Clinical outcomes

Clinical outcomes of first MTP joint fusion are generally favorable, with high rates of pain relief and patient satisfaction. A systematic review by Roukis [6] reported fusion rates of over 90% and significant improvements in pain and function. Hoveidaei et al. [7] reported that patients experienced substantial pain relief and improved walking ability postoperatively. Long-term outcomes have also been positive, with studies indicating sustained benefits over a decade [8-10]. 

Complications

Despite the high success rates, complications can occur. Nonunion, defined as failure of the bones to fuse, is one of the most common complications, with rates reported between 5-10% [11]. Other complications include hardware irritation, infection, and transfer of metatarsalgia. Nonunion is often managed with revision surgery, which may involve bone grafting or the use of advanced fixation techniques. Hardware irritation can often be addressed by hardware removal once fusion is confirmed [12]. 

Advanced fixation methods

Recent advancements in surgical techniques aim to improve fusion rates and reduce complications. Biomechanical studies have demonstrated that newer fixation methods, such as low-profile locking plates, offer superior stability and may reduce the incidence of hardware-related issues [13-15]. The use of biologic agents like bone morphogenetic proteins (BMPs) and autologous bone grafts have shown promise in enhancing bone healing and reducing nonunion rates. Additionally, minimally invasive techniques are being explored to reduce soft tissue dissection and improve recovery times [16-18].

## Materials and methods

Study overview

This retrospective study reviewed all patients who underwent their first metatarsophalangeal joint (MTPJ) fusion using continuous compression implants (CCI) between March 2020 and April 2024 at Bradford Royal Infirmary. A senior surgeon performed procedures. Patient data were collected from the surgeon's logbook, and a comprehensive review of medical records was conducted to gather demographic information, follow-up duration, and complications.

Ethical considerations

This study was conducted as a retrospective review using anonymized patient data, and as such, it did not require formal ethical approval in accordance with the policies of Bradford Royal Infirmary. Additionally, informed consent was not applicable as no patient-identifiable information was collected or used during the study.

Sample size calculation

This study included 27 patients (n=36 feet), reflecting all eligible cases during the specified time frame. Due to the retrospective nature of the study, a formal sample size calculation was not performed, and all consecutive patients meeting inclusion criteria were analyzed.

Study criteria

Patients who underwent primary first metatarsophalangeal joint (MTPJ) fusion using continuous compression implants during the study period were included, while those with incomplete records, revision surgeries, or follow-up durations shorter than six months were excluded.

Procedure

**Figure 1:**
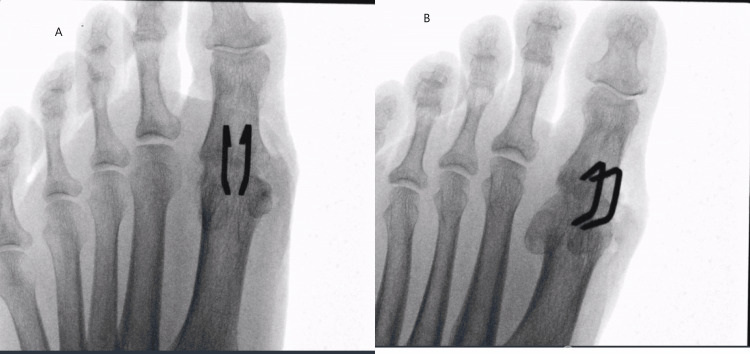
An intraoperative mini C-arm fluoroscope confirming the position of staples A: AP radiograph. B: Oblique radiograph AP: Anteroposterior

The procedure was performed under image intensifier guidance with the patient in a supine position. General anesthesia was administered, supplemented with an ankle block, and a thigh tourniquet was applied. A standard dorsal approach to the first MTPJ was utilized, and prominent osteophytes were excised. The joint surfaces were prepared using conical reamers. Initial stabilization was achieved using a 1.6 mm Kirschner wire (K-wire) passed centrally from the proximal phalanx into the MT head with manual compression of the bone. A flat metal foot tray was used to help simulate the weight-bearing position and confirm satisfactory positioning. Two parallel 15x12 mm Speed Depuy Synthes continuous action compression implants (staples) were implanted via the dorsal approach. An intraoperative mini C-arm fluoroscope was used to verify the satisfactory alignment, implant placement, size, and fixation quality of the MTPJ (Figure 1).

**Figure 2:**
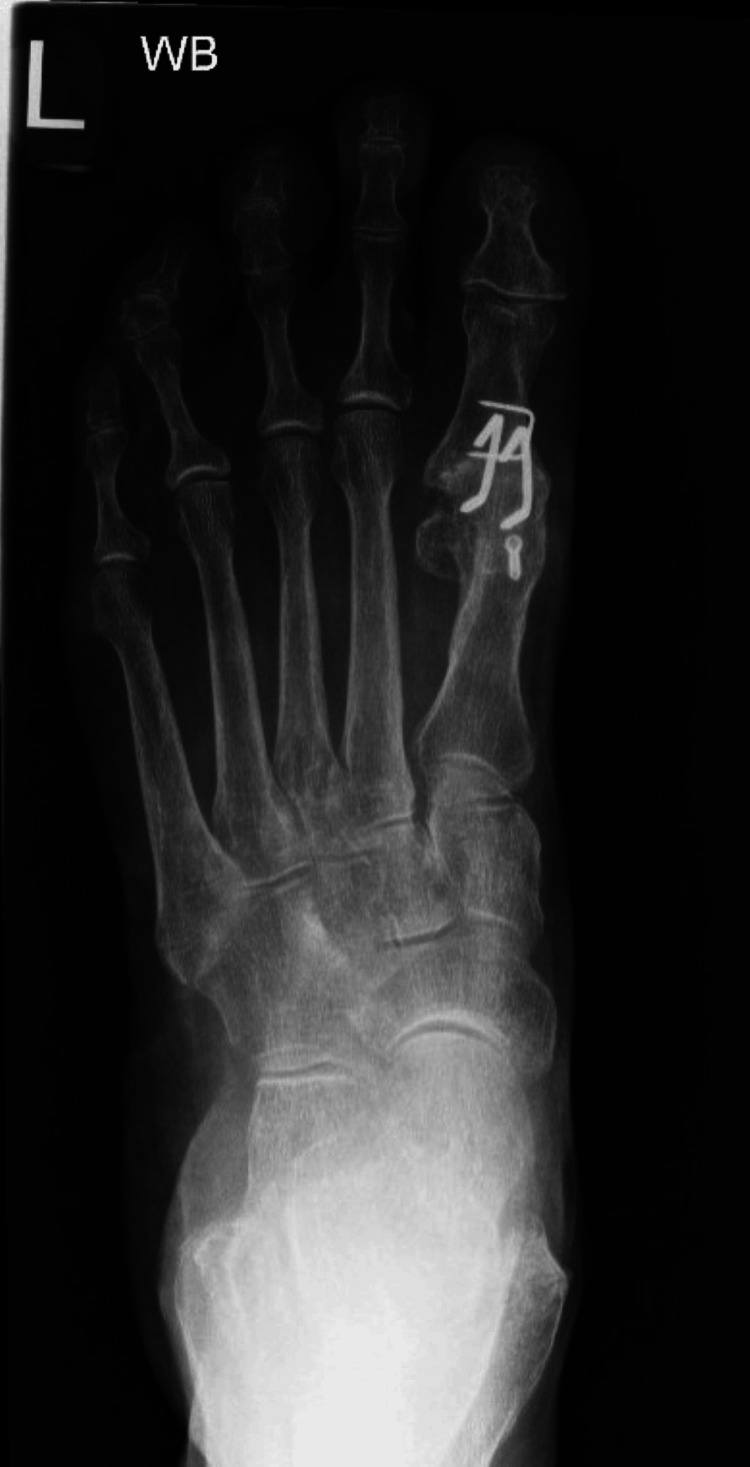
AP X-ray of left foot taken at six weeks showing signs of healing WB: Weight-bearing; AP: Anteroposterior

Postoperatively, patients were permitted to weight bear in a flat shoe +/- with the assistance of crutches for five minutes an hour for the first two weeks with patient-controlled weight-bearing durations after the two-week wound check until a review in clinic with radiographs at six weeks. If all was well, then the patient converted to no restrictions and their own footwear (Figure 2).

Tools of assessment

In this study, we used 15x12 mm Synthes staples, which are continuous-action compression devices. This is an extramedullary low-profile implant manufactured from Nitinol, a shape-memory metal, to continuously and dynamically keep bones compressed throughout the healing process, it comes with barbed legs that provide secure fixation and different lengths.

**Figure 3:**
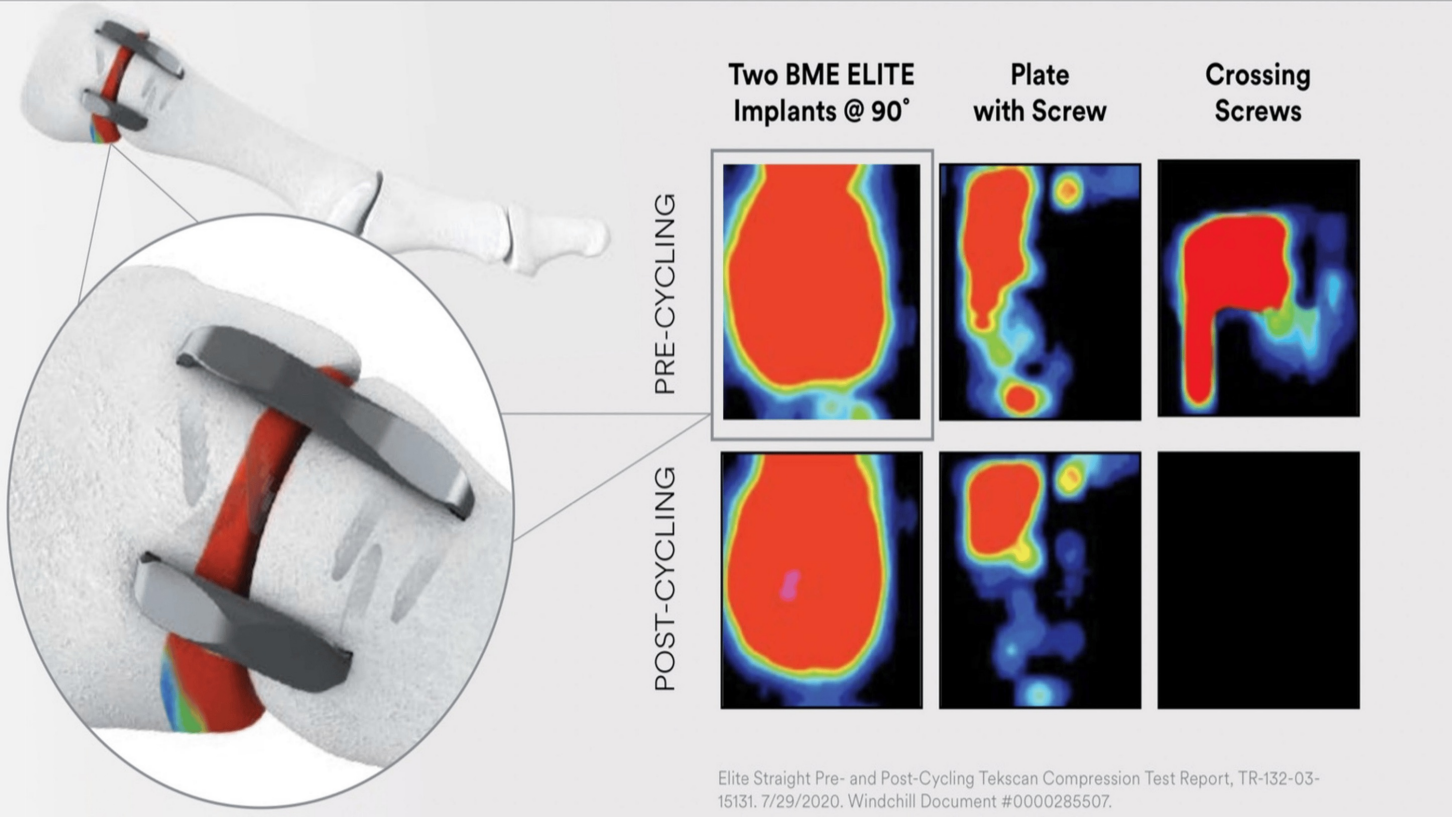
Illustration of biomechanical compression of stables in comparison to plates with screws and crossing screws. BME: Biomedical enterprises. Reprinted from the Comprehensive Overview of Orthopedic Solutions - CONTINUOUS COMPRESSION IMPLANTS (DePuy Synthes, 2021), with permission. Permission was obtained from DePuy Synthes.

Various CCI implants, such as the speed stable used in this study and the two BME Elite models depicted below; all operate based on the same fundamental principle of continuous compression. This mechanism not only aligns with but, in some instances, surpasses the performance of other designs in test cycling (Figure 3).

**Figure 4:**
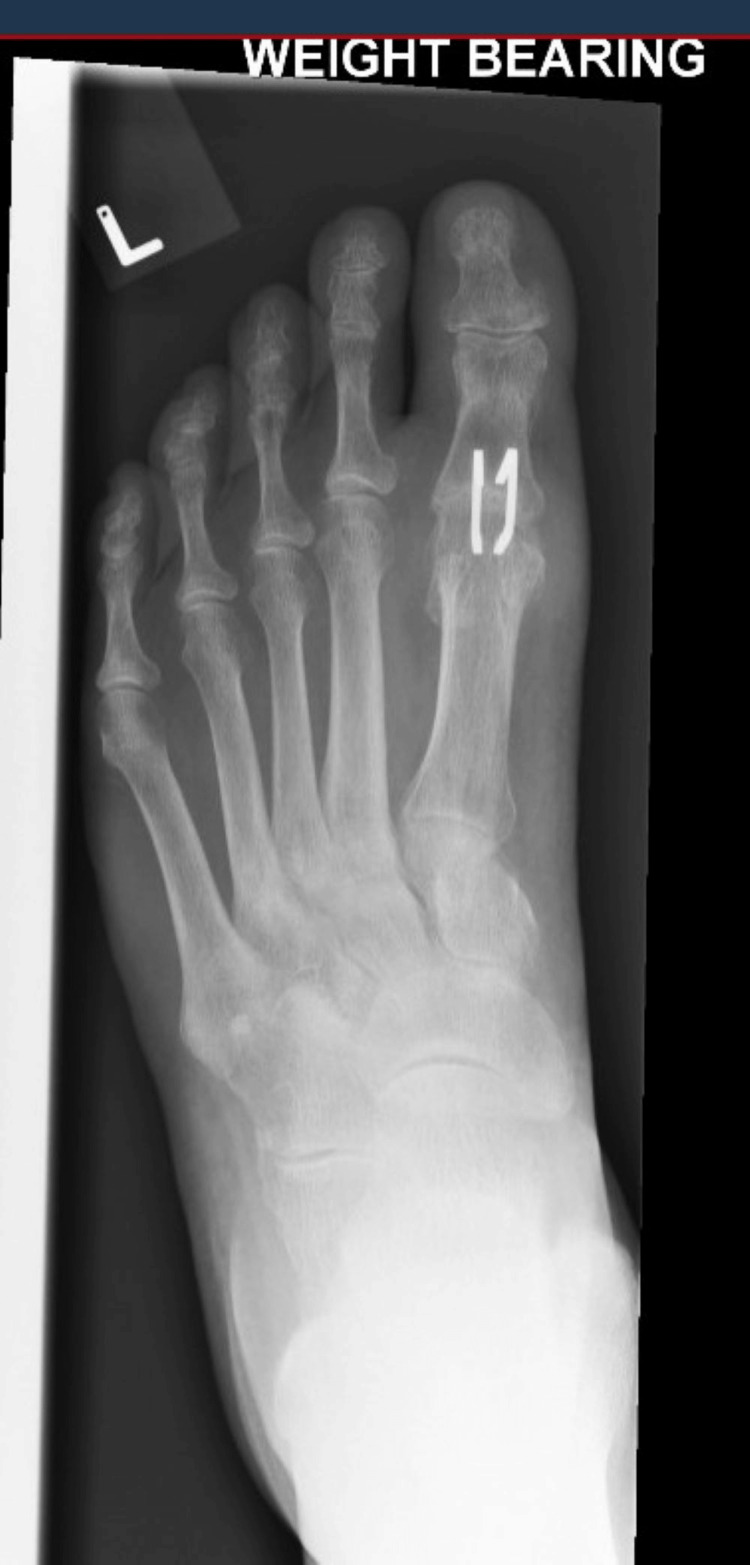
X-ray of left foot taken at three months showing healed fusion site.

Successful joint fusion was defined both clinically and radiologically. Clinical fusion was determined by the absence of pain during full weight-bearing and lack of tenderness or discomfort upon manual stressing of the joint. Radiological fusion was confirmed by the presence of trabeculae crossing the joint space in three out of four cortices (Figure 4).

Statistical analysis

Descriptive statistics were used to summarize patient demographics, follow-up duration, and complication rates. Results were expressed as mean (±SD) or median (range) values. Fusion rates were compared using appropriate statistical tests, with significance set at p < 0.05. Analyses were conducted using IBM SPSS Statistics software version 22.0.

## Results

A total of 27 consecutive patients totaling 36 feet who underwent primary fusion of the first MTPJ were included in this study (n=36). All procedures utilized the CCI device, and no cases were excluded. The mean age of the cohort was 60.24 years (range 41-90), comprising 24 females and three males. The distribution of left to right feet was 7 to 10, with nine patients undergoing bilateral fusion. Among the cohort, five patients had a background of inflammatory arthropathy requiring disease-modifying medications, two patients had type II diabetes mellitus on medication, and two patients continued smoking during the recovery period. The mean follow-up duration was 27 months (range 6-48 months).

**Figure 5:**
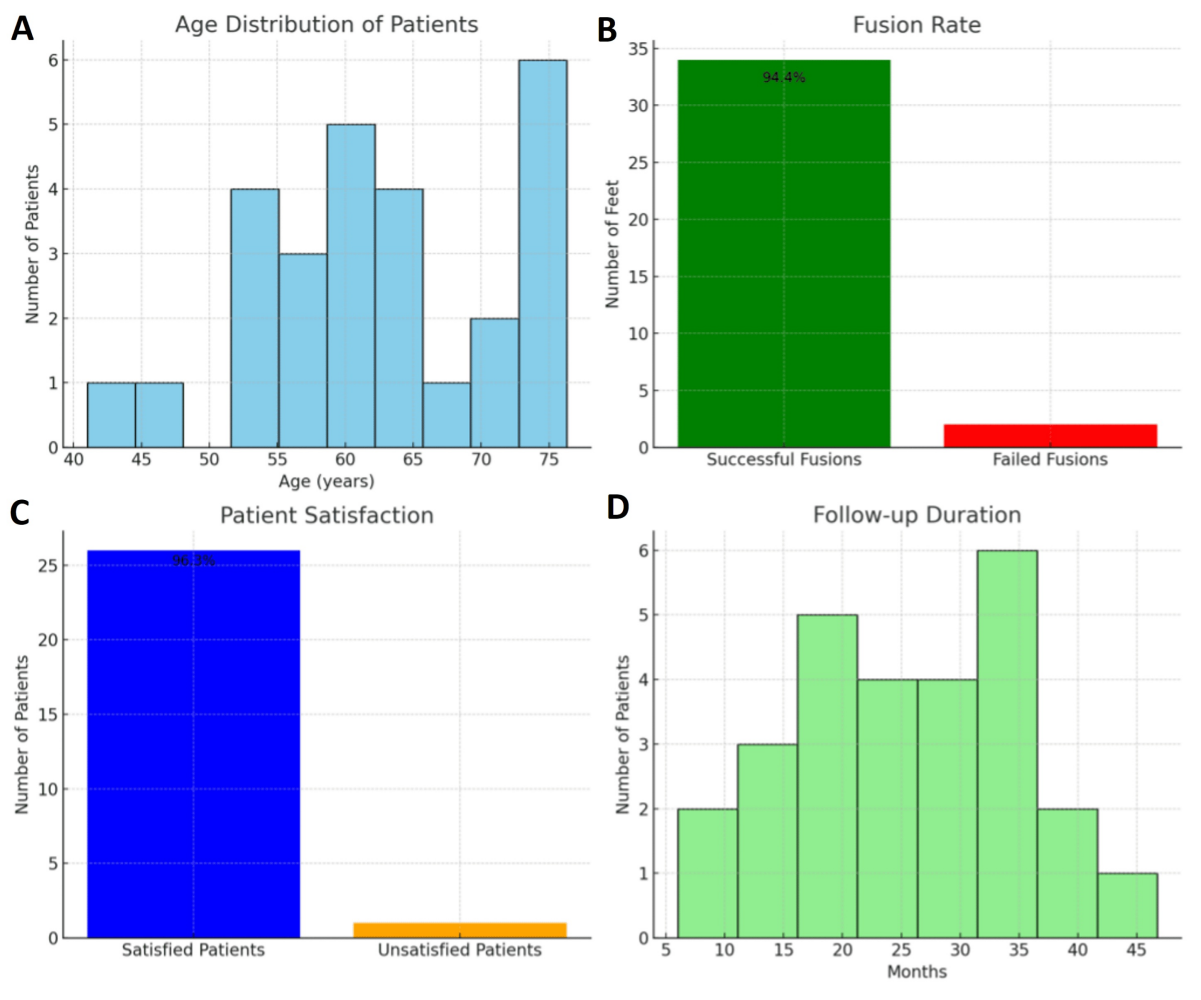
Graphical representation of patient factors and surgical outcomes in fusion procedures A: Age distribution of patients: Showing the age range and frequency of patients who underwent the procedure, indicating a concentration of cases among individuals aged 41-90. B: Fusion rate: Displaying the proportion of successful versus failed fusions, with a high success rate of 94.4%. C: Patient satisfaction: Illustrating satisfaction levels post-surgery, with a 96.3% satisfaction rate, indicating favorable patient-reported outcomes. D: Follow-up duration: Depicting the distribution of follow-up periods, showing that most patients were monitored between 6 and 48 months post-procedure.

Radiologically, fusion of the first MTP joint was achieved in 34 out of 36 feet (94.4% n=36) in 25 patients (92.6% n=27). 26 out of 27 patients (96.3% n=27) reported being either very pleased or fairly pleased with the overall outcome of the operation and indicated that they would be willing to undergo the same procedure on their other foot if symptoms arose (Figure 5).

**Figure 6:**
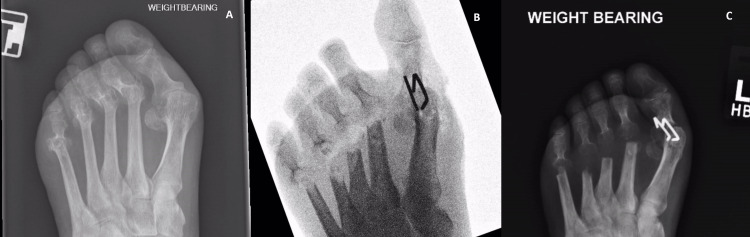
Serial X-rays of the left foot of a patient who underwent the first MTPJ fusion A: Preoperative X-ray; B: Intraoperative X-ray; C: Postoperative X-ray; MTPJ: Metatarsophalangeal joint

One of the early patients that ended with a nonunion and hallux valgus deformity was, in hindsight, an indication too far. Perhaps buoyed by early success, an MTPJ fusion with CCI was performed on a 56-year-old patient with rheumatoid arthritis with excision of the lesser metatarsal heads without change to the standard post-operative protocol. Images A, B, and C show DP radiographs pre-, intra, and post-operation at six weeks. Despite the nonunion and deformity, the patient was pain-free and declined my offer of revision surgery and remains as she is 18 months later. It is difficult to know whether a locking plate and/or weight-bearing restriction would have led to a union, but this case did help expose some limitations for CCI and confirmed that not all patients are suitable for this method. The author ensures a locking plate is available for all CCI MTPJ fusion cases and switches modes if the bone is significantly soft (Figure 6).

An interesting option may be to use a dorsal locking plate and a medial more plantar CCI instead of a screw, and technically would be straightforward to perform if the bridge distance between proximal and distal screws is measured on the plating system used.

## Discussion

The continuous compression device aims to achieve a robust fusion rate while minimizing the risk of subsequent hardware removal. This is facilitated by the device's ease of extraction, which reduces soft tissue irritation and the need for secondary surgical interventions [12]. The device delivers consistent compression across a broader surface area, enhancing the stability of the bony union and making it particularly advantageous in cases of suboptimal bone quality [15]. Our cohort achieved a fusion rate of 94.4%, comparable to rates reported with other implants used for this procedure.

Two CCI devices were chosen to allow a more uniform compression across a conical fusion site and to feel confident in allowing immediate protected weight bearing. With smaller feet, most commonly in females, there is not a lot of room, however, for two devices. This is overcome by placing the K-wire off center and placing the first CCI, and if there is restricted access for the second, removing the K-wire and proceeding with the second CCI. The construct is very stable with the single CCI, but the authors feel one single central CCI would potentially have more risks of failure than 2 dorsal CCIs. Care is needed to make sure the tips of the CCIs do not converge, and a parallel or divergent pattern is recommended.

Speed 1512 CCI was chosen for several reasons. The 15 is the length of the bridge and ensures that the limbs remain clear of the conical fusion site once they have been released from their holder and reverted to their compressive position. When stock levels have been problematic, 1512 and 1312 have been used as a pair, with the smaller one inserted second once most of the compression has happened. The 12mm legs have proven to be short enough not to pass through the plantar surface of the proximal phalanx in a problematic way while also providing enough hold in the bigger metatarsal head.

When performing a fusion for a conversion from a scarf osteotomy via a preexisting medial incision, there are a few choices to be made. Two dorsal CCI devices are still possible and have been achieved several times without having to remove the Akin staples of the distal scarf screw by the author. A medial, more plantar CCI and a dorsal CCI combination is also possible and has proved successful, though the experience of this is minimal.

If a CCI is removed due to malpositioning intraoperatively, it can be levered out with a small osteone or periosteal elevator. If this is delayed, the bone needs to be fully cleared before attempting it. The authors had one break on removal, and this was at the bridge limb joint. The limb was beneath the bone surface, and no retrieval was required.

Operative team is believed to be improved by the use of CCI over a plate screw construct. With sterile packed screws, it is a familiar story of guiding, drilling, measuring, and waiting for the screw to be found, checked, and handed to the surgeon. There is a clear time saving by preselecting and only using 1512 CCI. Because of the mix of indications (hallux valgus, hallux rigidus, and revisions) and extra procedures (PIPJ fusions and weils osteotomies), as well as the earlier cases being in a clear learning curve period, there was not enough data to prove a reduction in tourniquet time. The author believes there is a 15-minute saving between the two techniques. This comes from the footprint of the staples being quicker to prepare for a nice fit compared to a plate as well as less drilling (4 versus 6-7), no measuring, and no time spent fetching the screws. The drilling on the CCI system is via a guide with a hard stop, which is quick. Insertion is a push, release the handle and remove, and then a few hits with a tamp and hammer, which is again quicker than turning a screw.

In a like-for-like comparison with Synthes MTPJ plate at our hospital using our contract prices, the MTPJ fusion plate with six locking screws totals £321.85; if you chose to add a compression screw across the joint separate to the plate, this would add to the cost. Two CCI 1512 Speed Staples (Synthes) come to £247.54, and the single-use sterile instruments £54.38, totaling £301.92. It is possible to place the staples with the sterile instruments using (x2) 2.0mm K-wires and a punch and hammer for a small fragment kit, but you have to breach the plantar cortex each time to ensure the limbs won't bottom out, which isn't a worry with the instrument kits.

The authors offer caution when operating on osteoporotic bone. The author has one case where the intention was to use CCI, and the first device cut through the bone. This was in an elderly rheumatoid arthritic patient. The author has treated similar patients with CCI successfully but now ensures there is a plate and screw construct on standby and maintains his familiarity with the available system.

This study's limitations include its retrospective design, lack of a control group, and a relatively short follow-up period. Ideally, two years or more follow-up would be preferred to ensure that implant irritation does not manifest after the resolution of post-operative soft tissue swelling. However, the timing of implant removal may not be strictly related to follow-up duration. Wanivenhaus et al. reported a 20% rate of hardware removal after an average follow-up of one year with a dorsal plate and oblique neutralization screw [19]. In contrast, Chraim et al. did not report any hardware removals with a similar fixation method after an average follow-up of nearly four years [20].

## Conclusions

This study confirms the effectiveness of the first MTPJ fusion using the CCI device, with high rates of fusion, significant clinical improvements, and high patient satisfaction. This comes at a reduced cost compared to plate and screw constructs. The findings support using this technique as a reliable option for managing severe first MTPJ pathologies. Further studies with larger sample sizes and longer follow-up periods are recommended to validate these results and explore potential improvements in surgical techniques and post-operative. Once comfortable with their use, there would be a secondary benefit of shorter operation times as well.
